# Influence of Cu^2+^, Ni^2+^, and Zn^2+^ Ions Doping on the Structure, Morphology, and Magnetic Properties of Co-Ferrite Embedded in SiO_2_ Matrix Obtained by an Innovative Sol-Gel Route

**DOI:** 10.3390/nano10030580

**Published:** 2020-03-22

**Authors:** Thomas Dippong, Erika Andrea Levei, Iosif Grigore Deac, Emilia Neag, Oana Cadar

**Affiliations:** 1 Department of Chemistry and Biology, Technical University of Cluj-Napoca, North University Center of Baia Mare, 76 Victoriei Street, 430122 Baia Mare, Romania; dippong.thomas@yahoo.ro; 2 INCDO-INOE 2000, Research Institute for Analytical Instrumentation, 67 Donath Street, 400293 Cluj-Napoca, Romania; erika.levei@icia.ro (E.A.L.); emilia.neag@icia.ro (E.N.); 3 Babes-Bolyai University, Faculty of Physics, 1 Kogalniceanu Street, 400084 Cluj-Napoca, Romania; iosif.deac@phys.ubbcluj.ro

**Keywords:** cobalt ferrite, metal doping, sol-gel, nanocomposites, magnetic properties

## Abstract

This paper presents the synthesis of metal doped Co ferrites, M_0.2_Co_0.8_Fe_2_O_4_ (*M* = Cu^2+^, Ni^2+^, and Zn^2+^) embedded in SiO_2_ matrix by an innovative sol-gel route. The structural and morphological characterization provided information about the crystalline phases, crystallite size, and the shape of the prepared ferrites. The thermal study depicted the thermal decomposition and stability of the obtained ferrites. X-ray diffraction indicated nanocrystalline ferrites with spinel structure and the lack of crystalline phase impurities, while Fourier transform infrared spectroscopy revealed the presence of functional groups in precursors and ferrite powders. The lattice parameters showed a gradual increase indicating a uniform distribution of divalent metal ions in the Co ferrite lattice. The crystallite size, magnetic moment, super-exchange and deflection of magnetic domain were influenced by the dopant metal ion. The room temperature magnetization indicated a ferromagnetic behavior of the ferrites annealed at 1000 °C and a superparamagnetic behavior of the ferrites annealed at 700 °C.

## 1. Introduction

The MFe_2_O_4_ (*M* = Mn^2+^,Co^2+^, Ni^2+^, Mg^2+^, or Zn^2+^) type spinel ferrites have been raised as a novel group of versatile nanomaterials due to their tunable magnetic, electrical, and optical properties that makes them appropriate for an extensive range of applications, such as magnetic recording and sensing, information storage, catalysts, permanent magnets, transformer cores, radiofrequency circuits, waveguide isolators, gas sensors, hybrid supercapacitors, ferrofluids, inductors, converters, antennas, antibacterial agents, biocompatible magnetic-fluids, controlled delivery systems, and medical imaging techniques [1,2,3,4,5]. Xi et al. found that the saturation magnetization (*M_S_*) decreases with the increase in the transition metal ion content that substitutes Co^2+^ ions in the ferrites structure [2].

Cobalt ferrite (CFO) has an inverse spinel structure with Co^2+^ ions in the octahedral sites and Fe^3+^ ions distributed between tetrahedral and octahedral sites. CFO is a hard-magnetic material with remarkably high electrical resistivity, large permeability at high frequency, high coercivity (*H_c_*), moderate *M_s_*, high cubic magneto crystalline anisotropy, good mechanical hardness, and chemical stability [1,4,6,7]. The industrial applications of CFO depend upon its properties that are further determined by the preparation technique, grain size and structure, porosity, density, and cation distribution among the crystallographic lattice sites [1].

The doping with different transition or rare earth elements is an easy way to enhance the optical, electric, magnetic or biological properties. The total substitution of Co^2+^ by different cations (such as Ni^2+^, Cu^2+^, Mn^2+^, and Zn^2+^) within CFO results in enhanced strain sensitivity and resistive properties, as well as in the reduction in dielectric loss [8,9,10], while the doping in CFO leads in the improvement of electrical properties [11]. 

The optimal composition of doped CFO systems depends on the desired properties and is influenced by the cation distributions, variation in exchange interactions along with reduced anisotropy of the system due to preference of Co^2+^ ions for octahedral sites [10]. As the properties of spinel ferrites are greatly dependent on cationic distribution, the substitution with divalent ions can change the magnetic, thermal, and electrical properties [11,12,13]. The Co^2+^ substitution with Zn^2+^ in CFO affects the cation distribution and significantly alters the magnetic and magnetoelastic properties [9]. Recently, it has been shown that Zn-doped CFO with high strain sensitivity are potential materials for stress sensor applications, the magnitude of *H_C_* and magnetostriction decreasing with increasing Zn^2+^ content [3,4,14,15]. The Co^2+^ ions are generally responsible for the anisotropic contribution in CFO, while Zn^2+^ ions lead to enhanced dielectric and magnetic properties [10]. The doping with Cu^2+^ ions significantly changes the microstructure, crystal composition, particle size, and antibacterial activity [12]. The Ni^2+^ ions addition into CFO suppresses the grain formation, resulting in low surface roughness [8].

The broad range of potential applications of ferrites have boosted the number of researches dedicated to the development and exploration of different synthesis methods for nano-ferrites with specific properties (morphology, porosity, crystallite size, etc.) such as co-precipitation, microwave, electron-beam curing, sol-gel, citrate gel auto combustion, high-energy ball milling, thermal treatment, etc. [1,5,6]. Among these methods, the sol-gel synthesis followed by thermal treatment is a quite simple and inexpensive one as it requires a small number of chemical ingredients and a short production time compared to other synthesis routes [5].

Our previous studies presented the structural and magnetic changes of ferrites as a result of the variation of Co/Fe [13,16], Zn/Co [14,15,17], and Ni/Co [18] molar ratios. The present study investigates the changes in the structural, morphological, and magnetic properties of CoFe_2_O_4_ embedded in silica matrix, as a result of doping with low amounts of Zn, Ni, Cu (four times lower concentration than Co and 10 times lower than Fe), at different annealing temperatures (400, 700, and 1000 °C). The Zn^2+^ (Zn_0.2_Co_0.8_Fe_2_O_4_), Ni^2+^ (Ni_0.2_Co_0.8_Fe_2_O_4_), and Cu^2+^ (Cu_0.2_Co_0.8_Fe_2_O_4_) doped CoFe_2_O_4_ nanostructures embedded in SiO_2_ matrix were obtained using a sol-gel route from metallic nitrates, 1,4-butanediol (BD), and tetraethyl orthosilicate (TEOS). The comparative evaluation of X-ray diffraction (XRD) parameters (e.g. crystallite dimension, unit cell volume, lattice constant, X-ray density, physical density, and porosity) at 400, 700, and 1000 °C, offers important structural information on CoFe_2_O_4_ doped with transitional divalent metals. The thermal study (TG-DTA) depicts the formation and decomposition of metallic succinate precursors and the stability of the ferrites doped with Zn^2+^, Ni^2+^, and Cu^2+^ ions. The reduction in the vibration signal of the Co–O bond in CoFe_2_O_4_ as a result of doping and the enhancement of SiO_2_ matrix signals was followed by Fourier transform infrared spectroscopy (FT-IR). The evolution of magnetic properties (remanent magnetization (*M_r_*), *M_s_*, *H_c_*, magnetic moments per unit cell (*n_B_*), and anisotropy (*K*) with the increase in temperature and the doping effect of Zn^2+^, Ni^2+^, and Cu^2+^ were also investigated. 

## 2. Materials and Methods

### 2.1. Reagents

Nonahydrate ferric nitrate (Fe(NO_3_)_3_∙9H_2_O) of 98% purity, hexahydrate cobalt nitrate (Co(NO_3_)_2_∙6H_2_O) of 98% purity, hexahydrate nickel nitrate (Ni(NO_3_)_2_∙6H_2_O) of 99% purity, hexahydrate zinc nitrate (Zn(NO_3_)_2_∙6H_2_O) of 98% purity, trihydrate cooper nitrate (Cu(NO_3_)_2_∙3H_2_O) of 98% purity, 1,4-butanediol (BD) of 99% purity, tetraethyl orthosilicate (TEOS) of 99% purity, ethanol of 96% purity, and HNO_3_ 65% (Merck, Darmstadt, Germany) were used for the synthesis. 

### 2.2. Synthesis

CFO (60% CoFe_2_O_4_/40%SiO_2_), ZCFO (60% Zn_0.2_Co_0.8_Fe_2_O_4_/40% SiO_2_), NCFO (60% Ni_0.2_Co_0.8_Fe_2_O_4_/40% SiO_2_), and CCFO (60% Cu_0.2_Co_0.8_Fe_2_O_4_/40% SiO_2_) NCs were prepared through a sol-gel route using an M/Co/Fe molar ratio of 0.2/0.8/2, where M = Zn^2+^, Ni^2+^, or Cu^2+^. The sols were prepared by mixing the corresponding metal nitrates with BD, TEOS, ethanol, and HNO_3_. In all cases, a molar ratio of 1/1/0.67 NO_3_^-^/BD/TEOS was used. The resulted sols were mixed together under continuous stirring, for 30 min and kept in open air, at room temperature until gelation. The obtained samples were grinded, dried at 40 and 200 °C, thermally pretreated at 400 °C for 6 h, then annealed at 700 °C and 1000 °C for 6 h, as high purity gels with high crystallites size are obtained by using thermal pretreatment prior the annealing.

### 2.3. Characterization

The phase transformations that occurred during thermal decomposition were investigated by thermogravimetric (TG) and differential thermal analysis (DTA) using a Q600 SDT (TA Instruments, New Castle, DE, USA) simultaneous thermal analyzer, in air up to 1000 °C, at 10 °C /min heating rate and alumina standards. The formation and decomposition of functional groups was studied with a Spectrum BX II FT-IR spectrometer (Perkin Elmer, Waltham, MA, USA) on KBr pellets containing 1% (*w/w*) sample. The X-ray diffraction patterns were recorded using a D8 Advance diffractometer (Bruker, Karlsruhe, Germany), operating at room temperature, 40 kV and 40 mA with CuK*_α_* radiation (*λ* = 1.540,60 Å). The shape and clustering of nanoparticles were studied on samples deposited from suspension onto carbon coated copper grids using a HD-2700 (Hitachi, Tokyo, Japan) transmission electron microscope (TEM) with digital image recording system. The magnetic measurements were performed using a cryogen-free vibrating-sample magnetometer (Cryogenic Limited, London, UK). The hysteresis loops were recorded in magnetic fields between −2 to 2 T, at room temperature, while the magnetization *versus* magnetic field measurements were performed to find *M_S_* up to 10 T, on samples embedded in epoxy resin to prevent nanoparticle movement.

## 3. Results and Discussion

### 3.1. Thermal Stability

**Figure 1:**
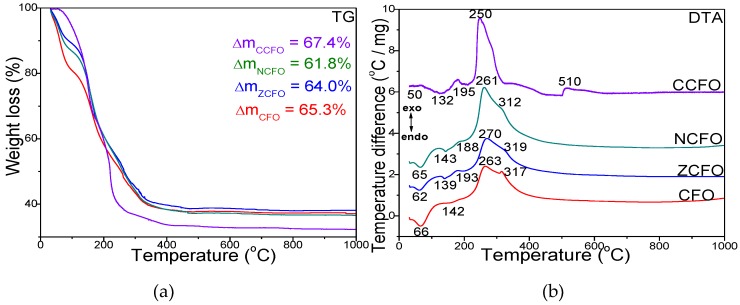
Thermogravimetric (TG) (**a**) and differential thermal analysis (DTA) (**b**) diagrams of CFO, ZCFO, NCFO, and CCFO dried at 40 °C.

Figure 1 displays the TG and DTA curves of CFO, ZCFO, NCFO, and CCFO samples dried at 40 °C. The DTA diagram of CFO sample shows three processes: (i) loss of physically adsorbed water indicated by the endothermic effect at 66 °C, (ii) formation of Fe- and Co-succinates indicated by the endothermic effect at 142 °C, (iii) decomposition of Fe- and Co-succinates indicated by two exothermic effects at 263 and 317 °C. The total mass loss of the process is 65.3%. 

The doping of CFO with Zn^2+^ and Ni^2+^ ions results in the appearance of a supplementary endothermic effect at 193 °C (ZCFO) and at 188 °C (NCFO) attributed to the formation of Zn- and Ni-succinates, respectively. Similar to CFO, the succinates decomposition takes place in two stages: (i) the decomposition of Fe- and Zn-succinates at 261 °C (ZCFO), of Fe- and Ni-succinates (NCFO) at 270 °C and (ii) the decomposition of Co-succinates at 312–319 °C. However, the exothermic effect attributed to the decomposition of Co-succinates in case of ZCFO and NCFO is not so well delimited as in case of CFO. As shown in the TG curves, the formation of ZFCO and NCFO takes place with a mass loss of 64.0% and 61.8%, respectively. When doping CFO with Cu^2+^ ions, the DTA diagram shows four processes associated with the loss of water, formation and decomposition of succinates. The physically adsorbed water loss (50 °C) and the formation of Co- and Fe-succinates (132 °C) take place at lower temperature compared with other ferrites, but the formation of Cu-succinate occurs at a higher (195 °C) temperature than the formation of Ni- or Zn-succinates in CFO doped with Ni^2+^ or Zn^2+^ ions. The decomposition of Fe-, Co-, and Cu-succinates appears as a single intense and wide exothermic effect at 250 °C. Compared with the other ferrites, a fourth process is indicated by the exothermic effect at 510 °C with no mass loss on the TG curve. This effect is attributed to the phase transformations that take place in the amorphous and weakly crystallized CCFO. The total mass loss of CCFO is 67.4%. Generally, compared with the undoped CFO, the CFO doped with Zn^2+^, Ni^2+^, and Cu^2+^ ions lead to the appearance of an additional endothermic effect attributed to the formation of Zn-, Ni-, or Cu-succinates and to the decomposition of Ni-, Cu-, Zn-, and Co-succinates in a single stage.

### 3.2. Fourier Transform Infrared (FT-IR) Spectroscopy

**Figure 2:**
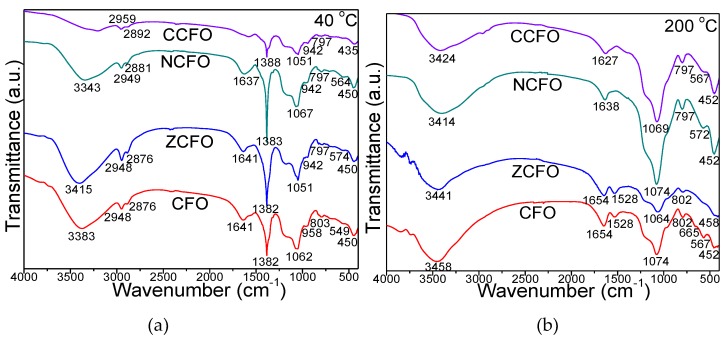
Fourier transform infrared spectra of CFO, ZCFO, NCFO, and CCFO samples heated at 40 (**a**) and 200 °C (**b**).

The FT-IR spectra of samples heated at 40 and 200 °C are shown in Figure 2. At 40 °C, the intense band at 1382–1388 cm^−1^ is characteristic to the nitrate group [7,13,14]. The disappearance of this band for samples heated at 200 °C confirms the complete reaction of nitrates and formation of succinate precursors. For samples dried at 40 °C, the bands at 2948–2959 and 2876–2892 cm^−1^ are attributed to asymmetric and symmetric stretching vibration of the C–H bond in BD, the band at 1637–1641 cm^−1^ to stretching and bending vibrations of O–H in BD and adsorbed molecular water, while the band at 3343–3458 cm^−1^ to O–H stretching and intermolecular hydrogen bonds in BD at 40 °C and in succinate precursor at 200 °C [13,14]. The band at 942–958 cm^−1^ is assigned to –OH stretching vibration and deformation vibration of Si–OH originated from the hydrolysis of –Si (OCH_2_CH_3_)_4_ (TEOS) [13,14]. 

At 200 °C, the bands at 1627–1654 cm^−1^ and 1528 cm^−1^ attributed to vibration of C=O of COO– groups, confirm the formation of a chelated complex by the coordination of carboxylate groups with metal ions [13,14,15]. In all spectra of samples heated at 40 and 200 °C, the absorption band at 549–574 cm^−1^ is assigned to the vibrations of tetrahedral M–O bonds and cyclic Si–O–Si structures, while the band at 435–458 cm^−1^ is attributed to the vibration of octahedral M–O and Si–O bonds vibration [16,17,18]. The band at 665 cm^−1^, attributed to the stretching vibrations of Co–O in CFO [13,14], disappears by doping with Zn^2+^, Ni^2+^, and Cu^2+^ ions. For samples heated at 40 and 200 °C, the presence of SiO_2_ matrix is indicated by the bands at 435–458 cm^−1^ (vibration of Si–O bond), 549–572 cm^−1^ (vibration of Si–O–Si cyclic structures), 797–803 cm^−1^ (symmetric stretching and bending vibration of Si–O–Si chains), 1051–1074 cm^−1^ with a shoulder around at 1200 cm^−1^ (stretching vibration of Si–O–Si bonds), 1627–1654 cm^−1^ (bending vibration of H–O–H bond), and 3343–3458 cm^−1^ (O–H bond vibration in the Si–OH group) [13,14,15,16,17].

**Figure 3:**
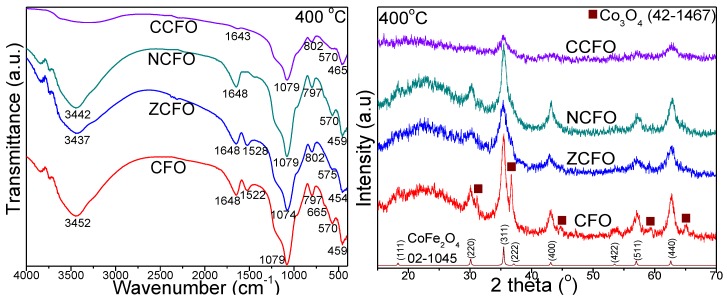

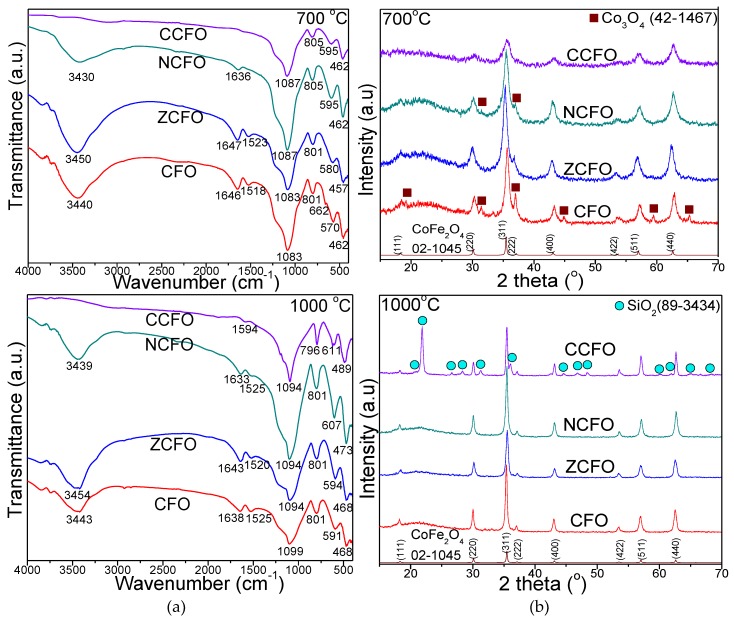
Fourier transform infrared spectra (**a**) and X-ray diffraction patterns (**b**) of CFO, ZCFO, NCFO, and CCFO annealed at 400, 700, and 1000 °C.

The FT-IR spectra of CFO, ZCFO, NCFO, and CCFO NCs annealed at 400, 700, and 1000 °C (Figure 3) show the presence of specific bands for SiO_2_ matrix: O–H bond vibration in the Si–OH group (3430–3454 cm^−1^), bending vibration of H–O–H bond at 1633–1648 cm^−1^ and 1518–1528 cm^−1^ (CFO and ZCFO), stretching vibration of Si–O–Si bonds (1074–1099 cm^−1^), symmetric stretching and bend vibration of Si–O chains in the SiO_4_ tetrahedron (796–805 cm^−1^), the vibration of Si–O bond (454–489 cm^−1^), and the vibration of Si–O–Si cyclic structures (570–611 cm^−1^) [13,14,15,16,17]. At 1000 °C, for CCF, the observed intensification of the characteristic bands of SiO_2_ is confirmed also by the presence of cristobalite according to XRD. The bands at 454–489 cm^−1^ and 570–611 cm^−1^ can be also attributed to the stretching vibration of octahedral M–O and tetrahedral M–O bonds, respectively, from the ferrites structure [13,14]. At 400 and 700 °C, for CFO an additional band at 662–665 cm^−1^ attributed to the formation of Co_3_O_4_ (identified by XRD) is observed.

### 3.3. X-ray Diffraction (XRD)

The XRD patterns of NCs annealed at 400, 700, and 1000 °C are presented in Figure 3. At 400 °C, CoFe_2_O_4_ (Joint Committee on Powder Diffraction Standards, JCPDS card no. 02-1045) has the highest crystallinity in CFO, but is contaminated with Co_3_O_4_ (JCPDS card no. 42-1467) [19]. The doping with Ni^2+^ ions favors the “purification” of ferrite, while the doping with Zn^2+^ or Cu^2+^ ions results in a lower crystallization degree of the ferrite. At 700 °C, the NCs are more crystalline resulting single phase ferrite by doping with Zn^2+^ (ZCFO) and Cu^2+^ (CCFO) and ferrite unpurified by Co_3_O_4_ by doping with Ni^2+^ (NCFO) or undoped CFO. At 1000 °C, the NCs are highly crystalline, for CFO, ZCFO, and NCFO, a single-phase cubic spinel structure with *Fd3m* space group was obtained, while for CCFO the ferrite is unpurified by cristobalite (SiO_2_—JCPDS card no. 89-3434) [19]. The intensity of diffraction peaks increases (the peaks became narrower and sharper) at higher annealing temperatures, due to the high degree of crystallinity and low effects of inert surface layer of the crystals [13,14,15,16,17]. The average crystallites size (*D*_XRDl_) was calculated using the Scherrer equation [17,18]. 

The crystallite size (*D_CS_*) calculated using the most intense diffraction peaks (311) for CFO, ZCFO, NCFO, and CCFO), unit cell volume (*V*), lattice constant (*a*), physical density (*d_p_*), X-ray density (*D_XRD_*), and porosity (*P*) for each NC calculated using the equations presented by Dippong et al. [18] are shown in Table 1.

Crystallite size is an important factor that influences the magnetic and optical properties of a material, especially when the grain size is nearing the crystallite size [6,18]. At 1000 °C, the crystallite size increases with increasing Cu^2+^ content, suggesting that the incorporation of Cu^2+^ enhances the grain growth in the formation of spinel phase [11]. The crystallite sizes also increase with increasing annealing temperature, as reflected by the narrowing and sharping of diffraction peaks [9,18]. This fact suggests that at high annealing temperatures (1000 °C) a significant agglomeration takes place without subsequent recrystallization, favoring the formation of a single crystal instead of a polycrystal [18].

The lattice constant shows an increasing trend with the decrease in crystallites size, while the *d*_XRD_ increases with increasing crystallite size. A variation of the lattice constant (*a*) and crystallite size (*D*_XRD_) as a result of different ionic radii, Fe^3+^(tetra: 0.49; octa: 0.64Å), Zn^2+^(tetra: 0.60; octa:0.74 Å), Cu^2+^ (tetra: 0.57; octa:0.73Å), Ni^2+^ (tetra: 0.58; octa: 0.69 Å), and Co^2+^(tetra: 0.58; octa: 0.74Å) was remarked [12,17]. The expansion of unit cell by the partial substitution of Co^2^ with Zn^2+^, Ni^2+^, and Cu^2+^ ions [20] could provide a possible explanation. The lattice constant (*a*) of CFO increases while the physical density *(d_p_*) decreases by doping with Zn^+2^, Ni^2+^, and Cu^2+^ ions. The rapid densification during the annealing process leads to the decrease of porosity (*P*) with increasing of annealing temperature [18]. The increase of *P* (from 9% to 19.3%, at 1000 °C) with decreasing physical density (*d_P_*) could be the result of the different grain size [3]. *P* of CFO increases only by doping with Cu^2+^, at 1000 °C, while for the other dopant ions (Ni^2+^ and Zn^2+^), *P* decreases, revealing that the obtained samples have highly dense structure.

### 3.4. Transmission Electron Microscopy (TEM)

**Figure 4:**
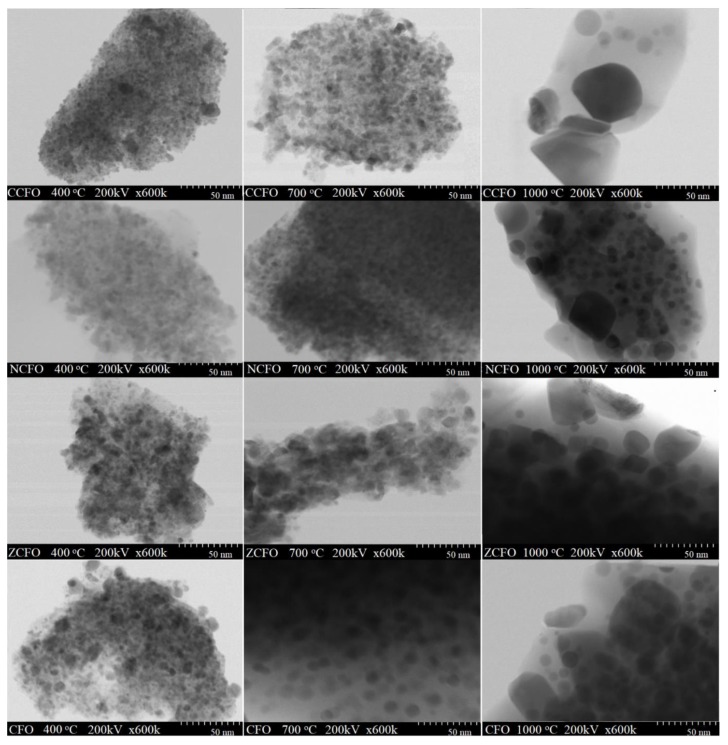
Transmission Electron Microscopy (TEM) images of CFO, ZCFO, NCFO, and CCFO annealed at 400, 700, and 1000 °C.

Figure 4 shows the shape and size of CFO, ZCFO, NCFO, and CCFO nanoparticles embedded in a SiO_2_ matrix annealed at 400, 700, and 1000 °C. For NCs annealed at 400 and 700 °C, the TEM images are blurry and the particles have irregular morphology, high tendency to form agglomerates and sizes ranging from 2 to 7 nm at 400 °C and from 6 to 14 nm at 700 °C. The high particle agglomeration is typical to chemically prepared NCs and is probably caused by the assembling tendency of very small particles [21]. By annealing at 1000 °C, a clear delineation of spherical particles is observed. Compared to CFO (32 nm), the doping with Cu^2+^ ions determine an increase of the particle size (47 nm), while the doping with Zn^2+^ and Ni^2+^ ions determine the decreases of the particle size (23 and 31 nm, respectively). The increase of nanoparticle size with increasing annealing temperature could be explained by the increase of crystal growth rate, volume expansion and supersaturation reduction of the system at high temperature [17]. Also, the internal heat energy produced during the thermal treatment causes an interfacial surface friction that lead to the agglomeration of particles [4].

### 3.5. Magnetic Properties

The magnetic measurements provide information about magnetic parameters such as *M_s_*, *M*_r_, and *H*_c_. The *M_s_* measurements were done in magnetic fields up to 10 T. In the absence of saturation, *M_s_* was estimated by fitting the magnetization vs. field curve *M(H)* [22]. The magnetic moments per formula unit (*n_B_*, in Bohr magnetons) and the multiaxial anisotropy constant (*K*) for nanoparticles were calculated using the equations presented by Dippong et al. [18] and are shown in Table 2.

**Figure 5:**
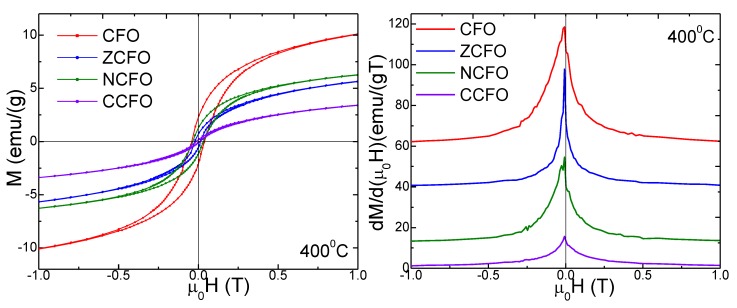

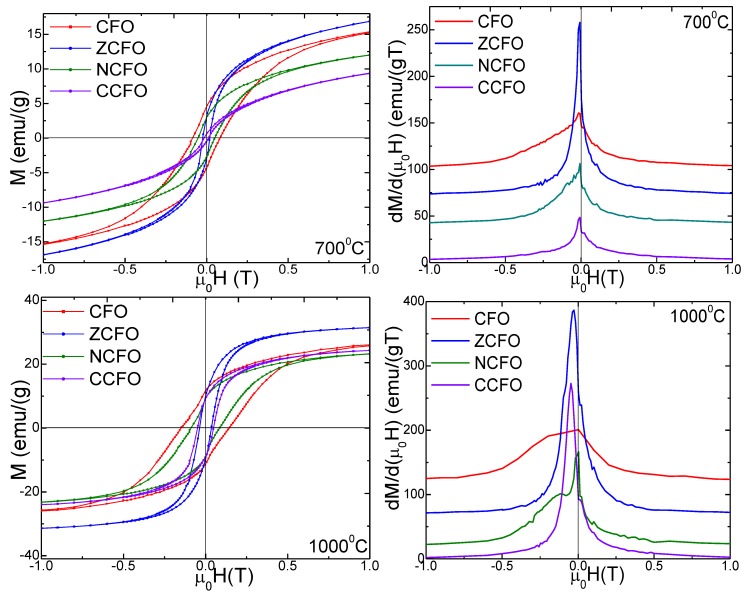
Magnetic hysteresis loops (**a**) and magnetization derivative (**b**) of CFO, ZCFO, NCFO, and CCFO annealed at 400, 700, and 1000 °C.

The magnetic hysteresis loops and the magnetization derivatives (*dM*/*d*(μ_0_H)) of CFO, ZCFO, NCFO, and CCFO NCs, annealed at 400, 700, and 1000 °C are presented in Figure 5. Most of our NCs show a superparamagnetic behavior with low *H_c_* values. At 400 °C, the largest magnetizations values were found for CFO, while the doping with Zn^2+^, Ni^2+^, and Cu^2+^ ions depreciate the magnetic properties. At higher annealing temperatures, the introduction of the non-magnetic Zn^2+^ ions in the Co-ferrite, leads to the increase of magnetization [23]. Almost all the derivatives of the hysteresis loops have small and broad peaks, indicating partially crystalline samples containing a large number of dislocations and other crystal defects. The peak height decreases with decreasing annealing temperature. 

For particles smaller than the critical diameter, the energy of the spin reversal is much lower than the thermal energy. In the absence of an applied magnetic field, the random orientation of the magnetic moments results in zero average global magnetic moment. In the presence of an applied magnetic field, a net magnetization occurs. The magnetic properties of the ferrites are strongly affected by the distribution of the cations and by the interactions of magnetic ions from inter- and intra-cells. In spinel ferrites, these interactions significantly affect the *M_s_* [11].

By doping CFO with Zn^2+^, the peak height of the hysteresis loop derivative significantly grows at higher annealing temperature, indicating an enhancement in the magnetic properties of ZCFO. A less evident improvement was remarked when CFO is doped with Cu^2+^ and annealed at 1000 °C. After annealing at 700 °C, two merged small peaks appear, indicating the presence of two magnetic phases. The doping of CFO with Ni^2+^ has no positive effect on its magnetic properties. The main magnetic parameters (*Ms*, *M_r_*, *Hc*, *n_B_*, and *K*) determined from the hysteresis loops *M(H)* are displayed in Table 2. The *M_s_* increases after annealing at 700 and 1000 °C for the Zn^2+^ doped Co-ferrite, while the *M_r_*, *H_c_*, and *K* lowers their values after doping with Zn^2+^, Ni^2+^, and Cu^2+^. A possible explanation could be the spin disorder created on the surface of ferrite nanoparticles, by the so-called surface effects [7]. The enhancement of CFO magnetization by doping with Zn^2+^ is well-known and could be explained by the preference of Zn^2+^ ions for tetrahedral sites in the crystalline spinel structure [7,9,23]. In this way, the Fe^3+^ ions from the tetrahedral sites are pushed in the octahedral sites increasing the magnetic moment in these sites [9,23]. As Zn^2+^ ions are not magnetic, this replacement will result in reducing magnetic moment at the tetrahedral site. Thus, since the overall sublattice magnetization increases, the net magnetization of NC will improve. The *M*_s_ value decreases with decreasing nanoparticle size as a result of the high surface to volume ratio, and of the associated surface effects [3,10,11,18]. The *H_c_* decreases by doping CFO with Zn^2+^, Ni^2+^, or Cu^2+^ ions as a result of higher spin disorder, especially at the surface layer, since *H*_c_ is significantly depreciated in smaller size particle where the spin disorder is increased [15,18]. 

The magnetic properties (including *Hc*) of the ferrite nanoparticles are strongly related to their sizes [16,17,24]. Below a certain size of the single magnetic domain nanoparticles, the *Hc* disappears and an ideal superparamagnetic behavior occurs. The *Hc* increases and reaches a maximum when the particles grow to a critical value to allow the formation of the magnetic multi-domains. For the NCs annealed at lower temperatures, containing Cu and Zn, the small size, single-domain particles are dominant in the particle size distribution, resulting in a superparamagnetic-like behavior (i.e., small *H_c_* and *M*_r_; not zero due to the presence of some larger size particles). For the NCs containing Co and Ni and for those annealed at 1000 °C, the mean particle size is larger and the magnetic behavior is ferromagnetic.

When the grain size attains the single domain state, the particles exhibit a superparamagnetic behavior and the surface effects become dominant over magnetization [6]. The *H_c_* decreases by CFO doping with Zn^2+^, Ni^2^, or Cu^2+^ due to the lower magneto-crystalline anisotropy of these ions when they replace Co^2+^ in the octahedral sites [5,10,18]. The lowest *K* value was obtained for CCFO at all annealing temperatures. The obtained results indicated that the difference between the magnetic properties of doped CFO depends on the dopant type, annealing temperature, crystallinity, particle size and shape. Also, an important contribution in the demagnetization process has the magnetic domain wall motion which can be affected by the decreasing particle size induced by the Zn^2+^, Ni^2+^, or Cu^2+^ ions doping [7].

## 4. Conclusions

The effect of Zn^2+^, Ni^2+^, and Cu^2+^ ions doping on the structural, morphological, and magnetic properties of CFO synthesized by modified sol-gel method was investigated. The doping leads to the appearance of a supplementary endothermic effect attributed to the formation of Ni-, Zn-, and Cu- succinates and a single exothermic effect attributed to succinates decomposition. The FT-IR spectra confirm the vibrational stretching modes of M–O bonds in the doped ferrites, at all annealing temperatures. At 400 and 700 °C, the XRD patterns reveal the formation of Co_3_O_4_ as secondary phase for undoped CFO and Ni^2+^ doped cobalt ferrite and of poorly crystallized ferrites for Zn^2+^ and Cu^2+^ doped Co ferrite. At these temperatures, the crystallite and particle sizes of doped Co ferrites are lower compared to those of undoped-CFO. At 1000 °C, the crystallite and particle sizes considerably increase, the largest values being obtained for the Cu^2+^ doped cobalt ferrite (47 nm). In the case of Zn^2+^ and Ni^2+^ doped Co ferrite, the particles and crystallites size (23 and 31 nm) have lower size than that of undoped Co ferrite (32 nm). However, except Cu^2+^ doped cobalt ferrite, the crystalline phases are pure. TEM images show the spherical structure of the nanoparticles at 1000 °C. The lattice constant, unit cell volume and physical density increase, while X-ray density and porosity decrease by doping with Zn^2+^, Ni^2+^, and Cu^2+^ ions. By doping CFO with non-magnetic Zn^2+^ ions, at high annealing temperatures, a significant enhancement of saturation magnetization results. The derivatives of the hysteresis loop curves suggest that the NCs are partially crystalline, having dislocations and other defects. The values of *Hc*, *M_r_*, and *K* decrease as a result of Zn^2+^, Ni^2+^, and Cu^2+^ doping. 

## Figures and Tables

**Table 1:** Particle size (*D*_TEM_), crystallite size (*D*_XRD_), lattice constant (*a*), unit cell volume (*V*), physical density (*d_p_*), X-ray density (*d*_XRD_), and porosity (*P*) of CFO, ZCFO, NCFO, and CCFO.

Temp (°C)	Sample	*D*_TEM_ (nm)	*D*_XRD_ (nm)	*a* (Å)	*V* (Å^3^)	*d_p_* (g/cm^3^)	*d*_XRD_ (g/cm^3^)	*P* (%)
400	CFO	7	6.8	8.385	590	4.654	5.283	11.9
	ZCFO	3	2.7	8.445	602	4.738	5.205	9.0
	NCFO	6	5.8	8.409	595	4.685	5.236	10.5
	CCFO	2	2.4	8.439	601	4.742	5.206	9.1
700	CFO	14	13.3	8.356	583	4.657	5.346	12.9
	ZCFO	10	9.6	8.372	587	4.738	5.338	11.2
	NCFO	9	8.9	8.379	588	4.752	5.299	10.3
	CCFO	6	5.8	8.395	592	4.790	5.285	9.4
1000	CFO	32	31.7	8.318	576	4.553	5.411	15.8
	ZCFO	23	22.6	8.344	581	4.597	5.393	14.8
	NCFO	31	30.8	8.323	577	4.561	5.400	15.5
	CCFO	47	46.3	8.300	572	4.412	5.470	19.3

**Table 2:** Saturation magnetization (*M_s_*), remanent magnetization (*M_r_*), coercivity (*H_c_*), magnetic moments per unit cell (*n_B_*), and anisotropy (*K*) of CFO, ZCFO, NCFO, and CCFO annealed at 400, 700, and 1000 °C.

Sample	*M_s_* (emu/g)			*M_r_*(emu/g)			*H_c_* (Oe)			*n_B_*			*K*·10^3^ (erg/cm^3^)		
	400	700	1000	400	700	1000	400	700	1000	400	700	1000	400	700	1000
CFO	14.8	22.4	28.7	2.6	4.7	12.1	400	880	1580	0.62	0.94	1.20	0.37	1.24	2.85
ZCFO	10.1	24.1	33.5	0.8	5.0	10.8	200	220	370	0.42	1.01	1.40	0.13	0.33	0.78
NCFO	9.65	18.2	25.2	1.4	3.2	9.5	360	540	900	0.40	0.76	1.05	0.22	0.62	1.42
CCFO	7.7	17.3	26.3	0.1	0.6	9.7	120	170	530	0.32	0.73	1.10	0.06	0.19	0.88
